# Modern strategies in classical fields of nanoindentation: Semiconductors, ceramics, and thin films

**DOI:** 10.1557/s43577-025-00923-w

**Published:** 2025-05-30

**Authors:** Xufei Fang, André Clausner, Andrea M. Hodge, Marco Sebastiani

**Affiliations:** 1 Institute for Applied Materials, Karlsruhe Institute of Technology, Karlsruhe, Germany; 2 Department of Microelectronic Materials and Nanoanalysis, Fraunhofer Institute for Ceramic Technologies and Systems IKTS, Dresden, Germany; 3 Mork Family Department of Chemical Engineering and Materials Science, University of Southern California, Los Angeles, USA; 4 Department of Civil, Computer Science and Aeronautical Technologies Engineering, Università degli Studi Roma Tre, Rome, Italy

**Keywords:** Nanoindentation, Electronics, Ceramic, Thin film, Fracture

## Abstract

Over the past three decades, nanoindentation has continuously evolved and transformed the field of materials mechanical testing. Once highlighted by the groundbreaking Oliver–Pharr method, the utility of nanoindentation has transcended far beyond modulus and hardness measurements. Today, with increasing challenges in developing advanced energy generation and electronics technologies, we face a growing demand for accelerated materials discovery and efficient assessment of mechanical properties that are coupled with modern machine learning-assisted approaches, most of which require robust experimental validation and verification. To this end, nanoindentation finds its unique strength, owing to its small-volume requirement, of fast-probing and providing a mechanistic understanding of various materials. As such, this technique meets the demand for rapid materials assessment, including semiconductors, ceramics, and thin films, which are integral to next-generation energy-efficient and high-power electronic devices. Here, we highlight modern nanoindentation strategies using novel experimental protocols outlined by the use of nanoindentation for characterizing functional structures, dislocation engineering, high-speed nanoindentation mapping, and accelerating materials discovery via thin-film libraries. We demonstrate that nanoindentation can be a powerful tool for probing the fundamental mechanisms of elasticity, plasticity, and fracture over a wide range of microstructures, offering versatile opportunities for the development and transition of functional materials.

**Graphical abstract:**

**Figure:**
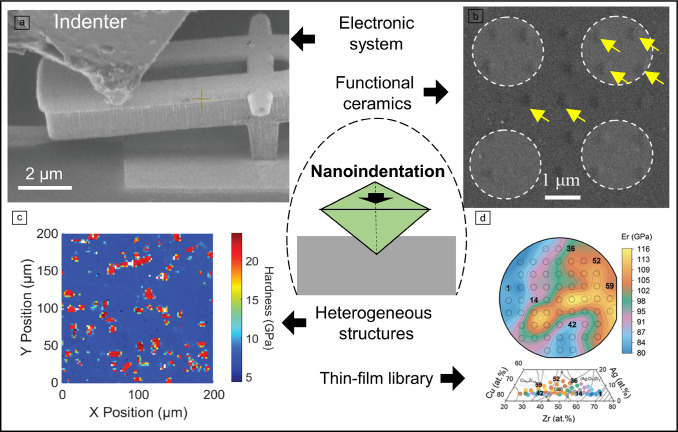
Modern strategies for nanoindentation in electronic systems, functional ceramics, heterogeneous structures, and thin films.

## Introduction

Since the seminal work by Oliver and Pharr in 1992^1^ on establishing the improved method for determining hardness and elastic modulus from indentation load–displacement data, nanoindentation has become a powerful and popular tool for versatile studies of the mechanical properties and materials physics, ranging from bulk metals,^1^ ceramic materials,^2^ thin films,^3,4^ polymers,^5^ biomaterials,^6^ battery materials, and composites. Nanoindentation is arguably one of the most popular mechanical testing techniques.^7^ The simplicity of sample requirement (small sample volume, well-polished surface), straightforward testing protocol, automated data collection and analysis, statistically available data set, fast testing speed, and modifiable testing stage/environment, to name a few, have significantly contributed to the success of this technique.

For more than three decades, nanoindentation has witnessed continuous development and transformation in materials characterization, transcending much beyond modulus and hardness measurement. Today, in response to the ever-increasing challenge in energy transition, which is essentially a transition of materials development, we are facing a rapidly growing demand for fast and robust assessment of materials mechanical properties with complex microstructures as well as for accelerated materials discovery and optimization. This relies, to a large extent, on efficient assessment and comprehensive understanding of the underlying mechanisms related to materials deformation and functionality. Meanwhile, with the miniaturization of functional/electronic devices and components, the limited materials volumes also call for reliable testing protocols for local targeted probing, where nanoindentation finds its playground.

Considering the large amount of literature available on nanoindentation and its versatile applications, it is not the intention of this work to cover all aspects such as high-temperature nanoindentation,^7^ low-temperature nanoindentation,^8^ modified nanoindentation loading frame for electrochemical charging, for example, hydrogen embrittlement studies,^9–11^ high-strain-rate nanoindentation tests.^12^ Instead, we will focus on a few emerging topics to highlight the wider applications of nanoindentation technique and demonstrate how this technique has evolved to meet new challenges in materials research. Specifically, this work features modern nanoindentation strategies by summarizing and discussing novel experimental protocols and perspectives for functional structure characterization, dislocation engineering in functional oxides, high-speed nanoindentation mapping, and accelerated materials discoveries via thin-film libraries. These examples demonstrate the nanoindentation method as a useful tool for probing the fundamental mechanisms in a wide range of structures and materials.

## Functional structure characterization in electronics

Modern microelectronic systems are complex heterogeneous and hierarchical multi-material systems. The size scales of the subsystems of the full electronic systems studied here, namely of the functional transistors on the semiconductor wafer and the on-chip interconnect stacks made currently mostly from brittle dielectrics and copper structures (the so-called back end of line, BEoL), are in the range of nanometers to a few micrometers. Therefore, all mechanical aspects related to the occurring thermomechanical–electrical interactions in these subsystems must be studied by means of micro- and nanomechanics. To experimentally study such effects, nanoindentation-based methods are best suited due to their initial simplicity and high resolution in force–displacement curves, but they need to be adopted for the specific tasks in electronics. Here, we focus on two different important nanomechanical aspects in electronics, strain-related parameter shifts in transistors as well as microcracking-related reliability risks. Both effects are mostly caused in real-life systems due to local micromechanical loads in the BEoL and the transistor device level induced by a thermomechanical interaction of the chip and its package, the so-called chip-package interaction (CPI).

**Figure 1:**
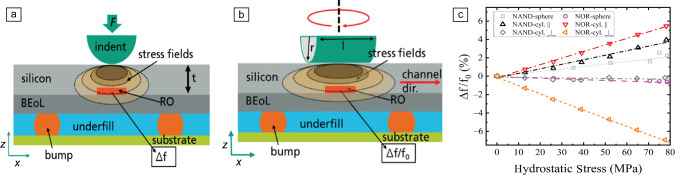
(a) Spherical nanoindentation approach to induce localized stress/strain fields into active ring oscillator (RO) structures in microelectronics.^14^ (b) Equivalent nanoindentation experiments, but using cylindrical tips in different orientations on active RO structures to test direction dependent strain effects on the electrical functionality in microelectronics.^15^ (c) Measured RO signal shifts (NAND and NOR ROs) for spherical and cylindrical indentation as a function of the (FE-derived) hydrostatic stress values at the ROs as well as the cylinder tip orientation caused by strain-induced charged carrier mobility shifts.^15^ Adapted with permission from References 14 and 15. © 2021 IEEE.

First, we focus on the influence of mechanical stress and strain on the performance of transistors. Local stress/strain directly affects the transistor device performance due to the piezoresistive effect of silicon in complementary metal oxide semiconductor (CMOS) technologies. This leads to a direct influence of strain on the charge-carrier mobilities in the transistor channels of n-type (NMOS) and p-type (PMOS) CMOS transistor variants.^13,14^ These effects have been experimentally assessed by nanoindentation-based methods using active ring oscillator (RO, each build up from 101 transistors)^13–18^ and static random-access memory (SRAM) memory cell (each build up from six transistors)^19,20^ test systems in different technology nodes. Indentation experiments have been performed on the backsides of thinned silicon wafers of the fully active electronic systems in close vicinity of the test structures (see **Figure** 1a).^14^ To induce high strains locally without mechanically cracking the brittle silicon wafer, spherical indentation tips have been used.^14^ To extract the stress–strain values at the transistor test structures, finite element method (FEM) simulations of the experimental setup have been exploited.^16,17^ To further increase the information content of the experimental data as well as to assess the anisotropy of the mechanical–electrical effects in the transistors (due to the anisotropy of the single-crystal silicon as well as the *n*- and *p*-well orientation of the CMOS technologies), cylindrical tip indentation experiments have been done (see Figure 1b).^13,15^ Using a combination of these spherical and cylindrical indentation experiments with electrical data of the active test structures and FEM simulations, the direction dependent mechanical-electrical effects could be derived for NOR-dominated and NAND-dominated ROs (NOR, NAND–basic logic functions build up differently using NMOS and PMOS transistors) including the piezoresistive tensors of the transistor channels (see Figure 1c).^15^

**Figure 2:**
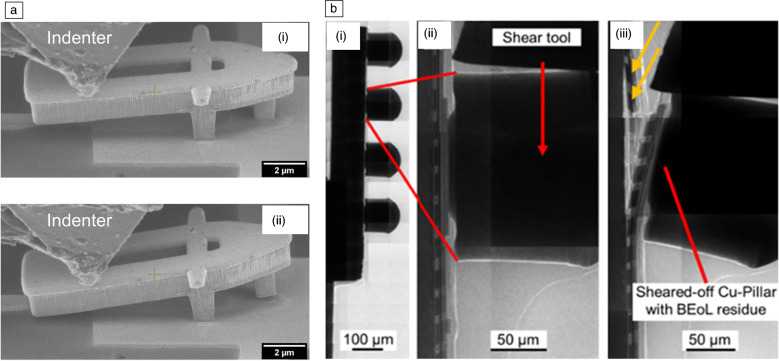
(a) Sequence of an *in situ* scanning electron microscopy nanoindentation cantilever push-to-pull test to quantify the interfacial adhesion in mode I (pull-off), (i) before and (ii) right after a delamination occurred.^21^ (b) *In situ* nano x-ray computed tomography lateral microindentation shear tip setup to inflict microcracking into Cu-pillar-BEoL stacks, with (i) the test vehicle in side view, (ii) condition before, and (iii) after the microshear experiment.^25^ Adapted with permission from References 21 and 25. © 2021 Elsevier; 2023 IEEE.

Second, we focus on the micro-/nanomechanical microcracking reliability of BEoL structures. To that, local adhesion failures of micrometer-sized interfaces have been studied using *in situ* SEM (scanning electron microscopy) nanomechanical experiments,^21,22^ and microcracking in full BEoL stacks has been studied using novel multidirectional indentation methods^23,24^ as well as *in situ* nXCT (nano x-ray computed tomography) lateral micromechanical shear experiments.^25^ Microcracking in BEoL stacks ultimately causes complete failures of the electronic system. This is getting more pronounced in recent technologies as those become smaller as well as more heterogeneously and mechanically stiffer integrated via, for example, Cu-pillars instead of softer solder bumps. In addition, as semiconductor substrates are getting thinner and therefore mechanically more flexible, which increases strain in the on-chip structures, electronics become increasingly prone to mechanical and thermomechanical failures. Resulting microcracking failures usually start very locally at a specific small BEoL interface and then increase in size and travel through the whole BEoL stack. Both failure stages have been studied using advanced indentation-based *in situ* nanomechanical testing procedures. To quantitatively test initial cracking stages very locally, micrometer-sized BEoL interfaces have been tested in terms of mode I failures (pull-off) using a novel cantilever test structure manufactured solely by the processes used for the BEoL production in real products. Those test structures have been tested using *in situ* SEM nanoindentation (**Figure** 2a) and the data have been evaluated using FEM.^21^ Similar experiments have been carried out for mode III failure modes (torsion).^22^ To further study later stages of multi-mode microcracking failures of complete BEoL stacks, novel micromechanical testing procedures implementing the detection of acoustic emission signals have been developed.^23,24^ And to gain even deeper insight into BEoL cracking, also *in situ* nXCT microshear experiments have been conducted using indentation-based shear experiments to inflict microcracking by laterally loading Cu-pillar level 1 interconnects inside an x-ray microscope, as demonstrated in Figure 2b.^25^

## Functional defects engineering in oxides

**Figure 3:**
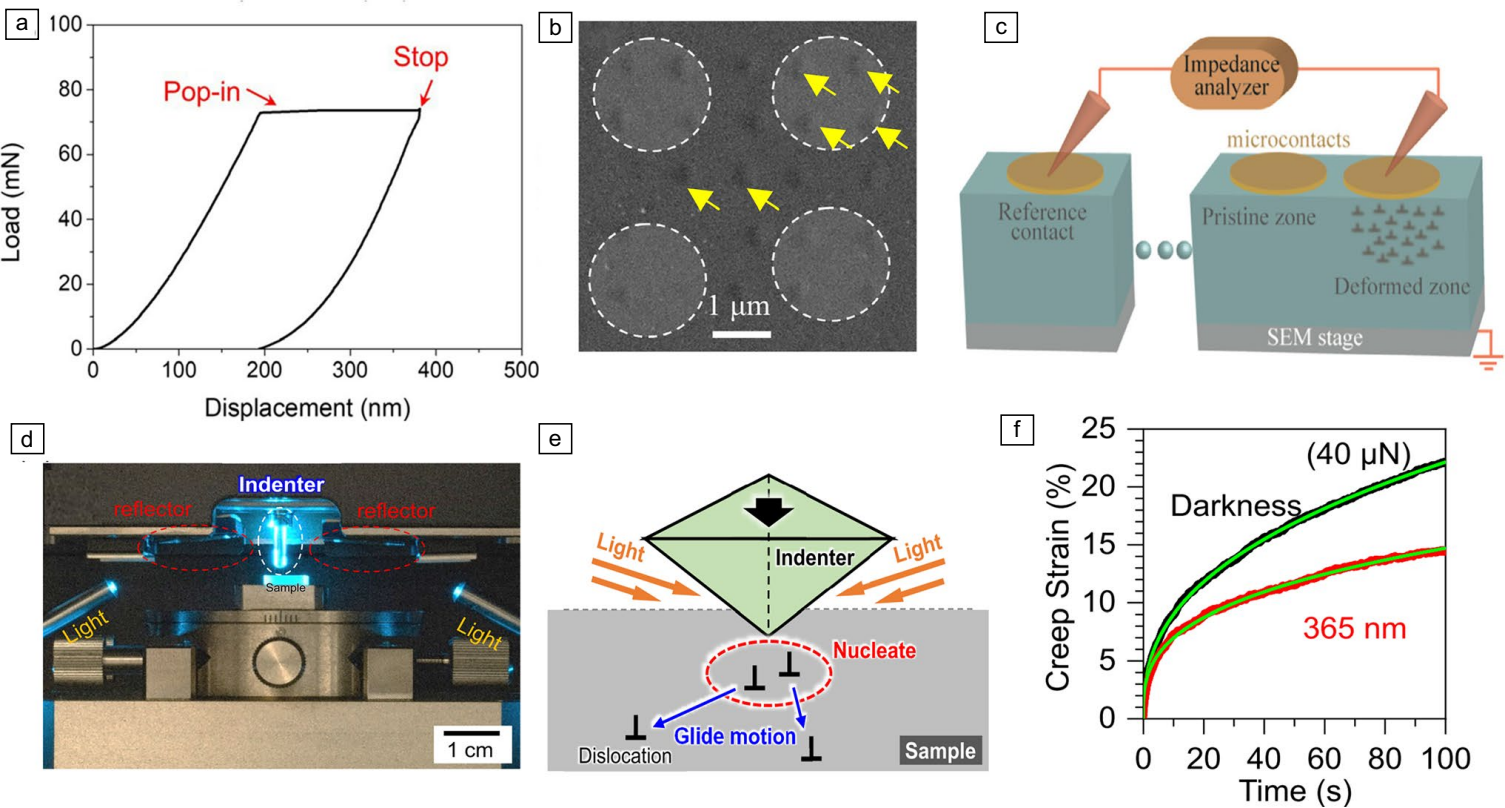
Novel application scenarios using nanoindentation tests: (a) Nanoindentation pop-in stop test^32^ (CC BY). (b, c) Nanomechanical imprinting of dislocation-rich regions for tunable electrical conductivity. SEM, scanning electron microscopy. Adapted from Reference 36 (CC BY-NC-ND). (d–f) Photoindentation for dislocation nucleation and motion subjected to darkness and various light wavelengths in compound semiconductors. Adapted with permission from Reference 37. © 2021 American Chemical Society.

Recent research has witnessed a renewed interest in dislocations in functional ceramics,^26–28^ with a focus on engineering dislocations to harvest the versatile functional properties such as superconductivity, electromechanical responses in ferroelectrics, electrical and thermal conductivities in thermoelectrics, as well as mechanical strength and toughness. Dislocations as line defects and the main carriers of plasticity may have been greatly underappreciated in the conventional picture of ceramics being brittle at room temperature.^28^ In order to mechanically imprint dislocations into functional ceramics, the strong ionic and/or covalent bonds must be broken under high shear stress while avoiding crack formation. In this aspect, nanoindentation is a most viable tool, where the nanoindentation pop-in event has been used as an indicator to probe the onset of plasticity. However, complications could arise due to the extremely high local shear force (as well as the hydrostatic compressive stress) in nanoindentation tests on ceramic materials. For instance, phase transition^29,30^ or cracking can be easily induced upon the pop-in events. Without proper control, dislocation pile-up underneath the indenter can eventually lead to crack initiation.^31^ Therefore, it is relevant to understand the incipient deformation process for better control of dislocation imprinting in ceramics. To this end, Fang et al.^32^ have focused on the nanoindentation pop-in stop tests (**Figure** 3a, in a load-controlled system) and developed an experimental and theoretical framework for achieving dislocation plasticity while suppressing the crack formation during nanoindentation pop-in tests of various oxides, for instance, SrTiO_3_, Al_2_O_3_, BaTiO_3_, and TiO_2_. This approach also found experimental validation on MgO^33^ and ZnO,^34,35^ to name a few.

These pop-in studies in ceramics have led to the identification of another size effect, in addition to the established indentation size effect in hardness^38^ and the pop-in size effect for incipient plasticity in metals.^39^ This new size effect in ceramics addresses the competition between plasticity and cracking during nanoindentation pop-in.^32^ With a sharper indenter (e.g., Berkovich indenter), the pop-in leads to dominantly dislocation plasticity in the crystalline oxides studied, while larger indenter tip size (e.g., 5-μm spherical tip on SrTiO_3_) normally leads to simultaneous cracking and dislocation plasticity.^32^ By circumventing crack formation using the optimized nanoindentation testing parameters, this new testing protocol has evolved as an effective mechanical imprinting technique for dislocation engineering in various functional oxides, providing a new path for functionality evaluation. For example, Bishara et al.^36^ programmed an array of shallow nanoindentation imprints (Figure 3b–c), which are rich in dislocations but free of cracks, on rutile TiO_2_ using a sharp Berkovich indenter and the pop-in stop test, and successfully measured an ~50% increase of electrical conductivity in the dislocation-rich region. This approach not only established a benchmark for using nanoindentation for nanomechanical programming of dislocation imprinting, but also opened many opportunities for fast screening of various functional ceramics for dislocation-tuned electrical properties, as further validated on yttria-stabilized ZrO_2_ for dislocation-enhanced oxygen ionic conductivity.^40^

Besides tuning the functional properties in advanced ceramics, coupling nanoindentation pop-in tests with external physical fields (e.g., light illumination for photoplasticity,^41^ electric field for electroplasticity^42^) or directly via defect chemistry engineering^43,44^ to evaluate the incipient plasticity in compound semiconductors has gained its momentum for various application-relevant scenarios. For instance, nanoindentation has found its unique strength investigating the dislocation mechanics under light illumination, coined as *photoindentation*^37^ (Figure 3d–e). This method has great potential in probing the dislocation nucleation via pop-in tests and dislocation motion by nanoindentation creep (Figure 3f). The very first photoindentation study was performed on single-crystal compound semiconductor ZnS,^37^ which exhibits one of the most significant photoplastic effect,^45^ under controlled light illumination conditions (from complete darkness to UV light with a wavelength of 365 nm, which is near the bandgap of ZnS). The nanoindentation tests suggest that light illumination has marginal impact on the dislocation nucleation but strongly hinders the dislocation motion, which is consistent with the bulk creep test results.^46^ The excellent agreement among the studies on dislocation motion across the length scale suggests that photoindentation, namely nanoindentation coupled with light illumination, is feasible for understanding the dislocation mechanics in compound semiconductors, without the need for testing large bulk crystals which can be extremely challenging and expensive to fabricate. The photoindentation technique has been recently extended to ZnO,^35,47^ another oxide that is sensitive to light illumination. It is expected that this technique will find its strength for high-throughput testing of materials once programmed with high-speed nanoindentation mapping.

## High-speed nanoindentation mapping

**Figure 4:**
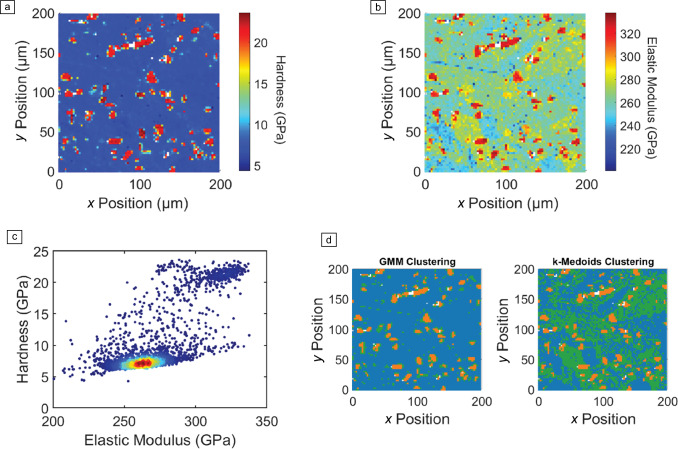
High-speed nanoindentation mapping of Stellite 6B hard metal (composition: Co base, 28–32% Cr, 3.5–5.5% W, 0.9–1.4% C). (a) Hardness map. (b) Elastic modulus map. (c) Density scatter plot of hardness versus elastic modulus, highlighting two primary clusters: hard carbides and the cobalt matrix binder. (d) Comparison of clustering methods: statistical-based Gaussian mixture model (GMM) and unsupervised *k*-medoids clustering. The *k*-medoids method identifies three key phases: blue for the cobalt matrix, orange for carbides, and green for the γ-phase of the cobalt matrix (face-centered-cubic (fcc) structure). The GMM clustering, in contrast, fails to distinguish the γ-phase.

High-speed nanoindentation mapping (HSNM), as its name suggests, is capable of rapid assessment of mechanical property distributions (e.g., hardness and elastic modulus) with unprecedented spatial resolution and field of view. This method is revolutionizing the characterization of heterogeneous functional materials (see **Figure** 4). Unlike traditional quasistatic nanoindentation, which requires significant time and delivers limited spatial data, HSNM performs an entire indentation cycle—including approach, contact detection, loading, unloading, and relocation—within one second. This capability facilitates high-resolution mapping over large areas, providing an unprecedented combination of speed and data density.^48^

Despite its advantages, implementing HSNM comes with specific challenges. Achieving consistent indentation depth and spacing is critical, particularly in materials with significant surface roughness or heterogeneity. For instance, the spacing between indents must adhere to criteria such as 10 times the depth to avoid interactions between adjacent plastic zones.^49^ High strain rates inherent to HSNM can affect materials with strain rate sensitivity, such as polymers and superplastic metals, potentially leading to deviations in mechanical property measurements.^50^

Another challenge involves the reliability of data interpretation. Heterogeneous systems, such as battery composites or cementitious materials, often exhibit artifacts like porosity or interfacial delamination. Advanced filtering and data analysis techniques, such as statistical deconvolution or even machine learning methods, have been developed to minimize noise, detect outliers, and interpret the mechanical phase distribution.^51,52^ Additionally, maintaining the sharpness of indenter tips during thousands of cycles is crucial for ensuring consistent measurements across extensive mappings.^53^ A recent review article on this subject^48^ describes in detail the main experimental issues and the criticalities in the adoption of advanced data analysis tools.

HSNM has become indispensable in exploring complex material systems with diverse applications:

- *Additively manufactured metals:* HSNM has been instrumental in phase identification and mechanical property mapping of multiphase alloys. Correlations with electron backscatter diffraction (EBSD) have revealed grain orientation-dependent behavior, aiding the development of high-performance alloys for aerospace and automotive sectors.^48^
- *Battery composite materials:* In Li-ion battery composites, HSNM has been used to distinguish matrix-reinforcement properties, detect defects, and optimize mechanical performance, significantly advancing energy-storage research.^54^
- *Cementitious systems:* HSNM has enabled the differentiation of high-density (HD) and low-density (LD) calcium silicate hydrates (C–S–H), providing critical insights into hydration processes and durability in construction materials.^55^
- *Natural and bioinspired materials:* The technique has facilitated the investigation of hierarchical architectures in nacre and bone, uncovering the mechanical strategies that inspire next-generation biomimetic designs.^56^

Worth noting is that, HSNM offers versatile possibilities beyond simple mapping of hardness and elastic modulus. A recent study has demonstrated the simple use of optical profilometry, instead of adopting consuming atomic force microscopy (AFM) area measurement, to correct for pile-up effects for large arrays of indents.^57^ This would be of particular interest for semiautomated correction of pile-up errors over individual phases, when performing high-speed maps on multiphase materials. Additionally, the HSNM is also suitable for spatially resolved measurements of fracture toughness in strongly heterogeneous ceramics (e.g., by using the pillar splitting geometry)^58^ that allows for site-specific fracture toughness assessment and is fully compatible with the high-speed testing mode. In this way, as an example, the fracture toughness can be mapped across the complex microstructure of thermal barrier coatings, or even the fracture toughness of thermally grown oxide (TGO) can directly be mapped across the interface between the top-coat and the bond-coat.^51^ It is expected that HSNM will become a powerful tool not only for fast testing, but also for accelerated materials discovery.

## Accelerated materials discovery via thin-film libraries

**Figure 5:**
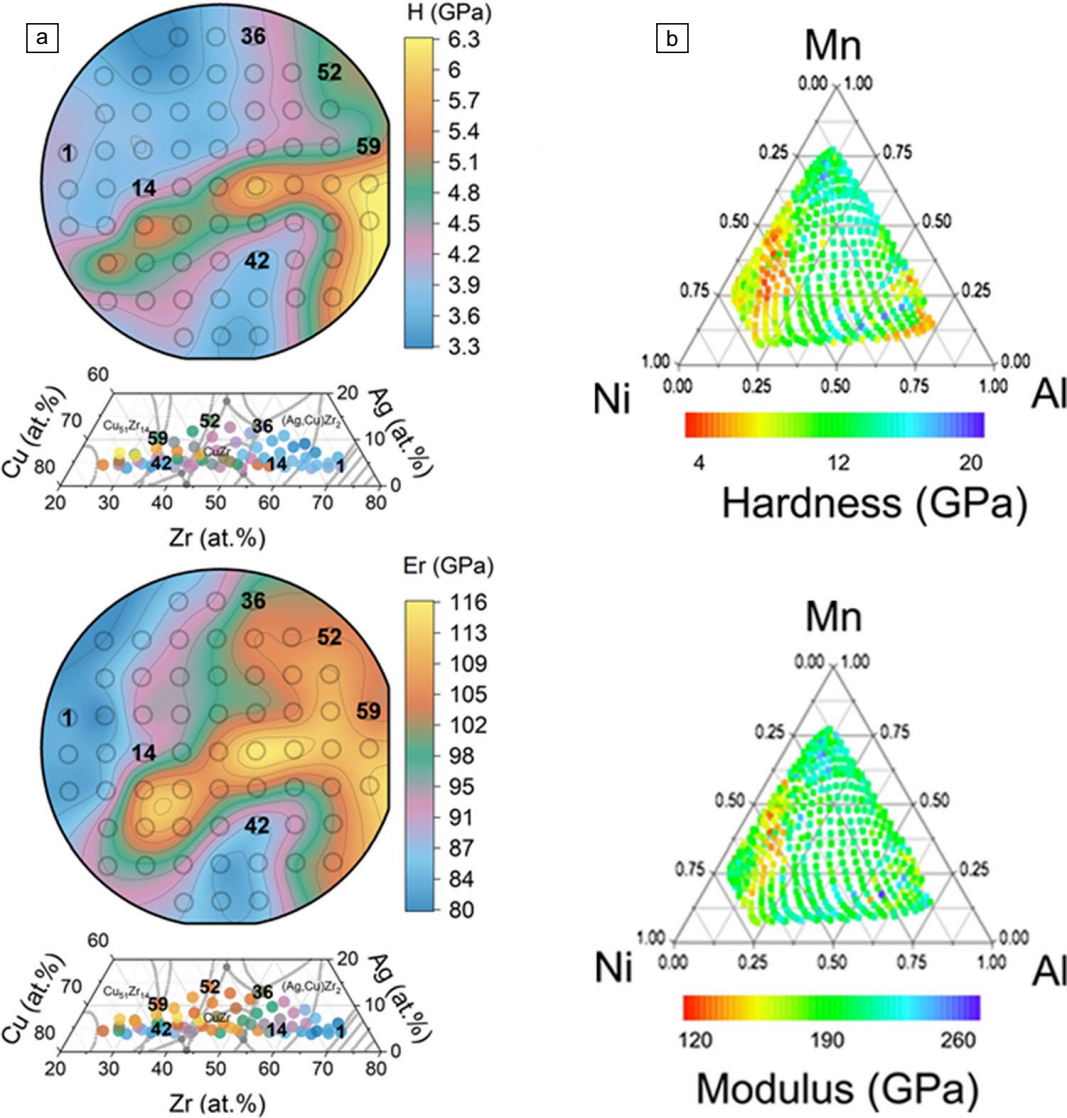
Examples of nanoindentation thin-film libraries showing (a) contour plots from 61 CuAgZr compositions. Adapted with permission from References 73 and 74. © 2008 AIP Publishing; 2023 Wiley. (b) Ternary diagram from 535 NiMnAl compositions. Reprinted with permission from Reference 73. © 2008 AIP Publishing.

Accelerated materials discovery has gained momentum in recent years due to the onset of automation, fast production and screening, and the ability to manage large data sets via machine learning (ML) algorithms. Of particular interest is the thin-film libraries development. Thin-film libraries typically prepared via sputtered deposition are a subset of combinatorial synthesis techniques such as diffusion couples, and additively manufactured parts.^59–61^ These methodologies enable the simultaneous and rapid production of tens, hundreds, or even thousands of individual samples by generating compositional gradients and thus compositional libraries. Furthermore, material libraries (MatLibs) combined both combinatorial synthesis with high-throughput characterization techniques to enable fast screening of large databases and generate composition–structure–property relationships, including microstructural, thermal, and mechanical behavior.^62–72^ In the context of high-throughput property mapping, nanoindentation stands out as a practical technique due to its minimal sample preparation requirements, and ease of automation to map a large number of samples, providing both hardness and modulus maps (**Figure** 5). The term combinatorial high-throughput MatLibs is in contrast to “mechanical mapping,” where single samples are mapped using fast and closely packed indents,^53^ as previously discussed in HSNM.

Although publications on combinatorial indentation can be traced back to the early 2000s,^75,76^ recent focus on accelerated materials discovery has led to renewed interest and new research. Nanoindentation MatLibs can provide an unprecedented amount of information for accelerated material design. However, there are several challenges that arise from the lack of standard library characterization. Notably, regardless of the material system and synthesis methods, the number of compositions and indents performed per library varies widely, where the number of compositions per “library” can vary from 3 to 500 and the number of indents per composition can range from 1 to 50.^73,77,78^ Additionally, integrating and understanding the large amounts of data generated from nanoindentation libraries remains challenging, as the implementation of artificial intelligence (AI) techniques is still in its infancy. Recently, ML models have been incorporated for training nanoindentation MatLibs in CuAgZr (see e.g., Figure 5a) and Pt-Au systems.^74,79^ However, in order to fully exploit AI techniques for nanoindentation libraries, new models such as time series forecasting should be developed in tandem with large and accessible databases.

A brief overview highlighting examples of nanoindentation MatLibs are presented here for a wide class of materials and compositions per library, including metallic alloys, ceramics, and polymers. In general, nanoindentation studies of binary and ternary alloys have been conducted to link mechanical behavior to compositionally driven structural changes, such as increased hardness from intermetallic formation in TiAl,^80^ nanotwinning in CuNi alloys,^81^ and precipitate formation in TiNiCu.^78^ Other studies have screened for simultaneous optimization of multiple properties, such as hardness and corrosion resistance in the Al-rich regions of a ZnAl library.^82^ For compositionally complex alloys, studies have explored the correlation between composition and changes in grain size, resistivity and hardness changes in CrMoNbTaVW,^83^ phase content and mechanical behavior in nanocrystalline in CoCrFeNi,^84^ and identifying promising high-ductility, high-strength regimes in the NiTiVZr system.^85^ As for ceramic systems, investigation of a SiFeCN material library identified compositions with improved biocompatibility, hardness and reduced modulus, which were linked to increased N and Si and decreased Fe content.^86^ Applicability to polymer samples is also reported in the study of an acrylate-based library that demonstrated that the mechanical properties of a given co-polymer can significantly deviate from the behavior of either of its constituents.^87^ Beyond thin-film approaches, combinatorial nanoindentation has been used in diffusion-couple based material libraries to identify shape-memory behavior and the dependence of the recovery temperature on composition of a TiPd-TiNi system.^88^ Additively manufactured material libraries include the characterization of Ni-content effects on the phase-fraction, microstructure, and mechanical behavior of TiNi,^89^ as well as the impact of increased Ti-content on the formation of Laves and Chi phases, which leads to hardening and embrittlement of CoCrFeNiTi.^90^

## Summary

The continuous development of the nanoindentation technique makes it an invaluable tool beyond its conventional application for modulus and hardness measurement. Nowadays, the nanoindentation technique has become an effective tool for assessing the performance and reliability of modern electronic systems, engineering tailored functional defects, mapping out the mechanical properties, as well as accelerating discovery of new materials. We have showcased several representative modern strategies of using nanoindentation in these aspects, covering semiconductors, functional ceramics, heterogenous functional materials, and compositionally complex alloy thin films.

By coupling with multiple fields such as thermo-chemo-mechanical–electrical effects at small scales, nanoindentation-based experimental strategies can be developed to realize reliability assessments as well as nanomechanical programming in semiconducting components and functional oxides. This significantly improves the understanding of these effects and ultimately leads to faster, more robust, and more reliable new generations of highly integrated electronic technologies. With the fast mapping and quantified nature for mechanical properties including modulus, hardness, and fracture toughness, nanoindentation is proven a powerful tool for probing the fundamental mechanisms involving elasticity, plasticity, and fracture over a wide range of materials components and complex microstructures. Last but not least, materials libraries combining multiple properties including nanoindentation data can serve as a pathway for future materials development. The large databases developed in materials libraries can be implemented into machine learning models to further expedite and explore accelerated materials design. It is clear that nanoindentation testing will continue its journey, beyond the Oliver–Pharr method, for advanced materials characterization and development.

## Data Availability

All data related to this work have either been made open access or can be shared upon reasonable request to the corresponding authors.
